# Chemical Diversity and Biological Significance of Sesquiterpene Lactones: From Biosynthesis to Anticancer Mechanisms

**DOI:** 10.1002/cbdv.71840

**Published:** 2026-10-22

**Authors:** Kedar Nimba Chaudhari, Ragini Yadav, Mayank Kashyap, Shreyans K. Jain

**Affiliations:** ^1^ Department of Pharmaceutical Engineering and Technology Indian Institute of Technology (BHU) Varanasi Uttar Pradesh India

**Keywords:** alantolactone, anticancer activity, biosynthetic pathway, parthenin, sesquiterpene lactones, *α*‐methylene‐*γ*‐lactone

## Abstract

Cancer is one of the world's leading causes of death, demanding novel therapies with potent and selective anticancer activity. Sesquiterpene lactones (SLs) are structurally diverse secondary metabolites derived from plants, predominantly found in the Asteraceae family, and are recognised for their extensive pharmacological significance. Characterised by a 15‐carbon skeleton and an electrophilic *α*‐methylene‐*γ*‐lactone moiety, SLs exhibit notable anticancer, anti‐inflammatory, and immunomodulatory activities. Relevant original research and review articles published between 1994 and 2026 were identified through scientific databases such as Science Direct and Google Scholar. The biosynthesis of SLs from isopentenyl diphosphate and dimethylallyl diphosphate via farnesyl diphosphate highlights enzymatic cyclisation and oxidation steps that generate distinct carbocyclic frameworks. Structure‐activity relationship studies emphasise the critical role of Michael acceptor functionalities in mediating biological activity through covalent interaction with cellular nucleophiles. Mechanistically, SLs exert anticancer effects by inhibiting NF‐*κ*B signalling, inducing oxidative stress, activating apoptotic pathways, causing cell‐cycle arrest, and modulating MAPK signalling. Recent advances in semisynthetic modification strategies aimed at improving potency and selectivity are discussed. Highlighting the need for more comprehensive pharmacokinetic, toxicological, and clinical investigations. Overall, SLs represent valuable, structurally versatile scaffolds for anticancer drug discovery, although further optimisation and clinical validation are required to establish therapeutic potential.

## Graphical abstract

Sesquiterpene lactones emerge as structurally diverse anticancer scaffolds with multifaceted mechanisms of action. Their biosynthesis, chemical diversity, structure–activity relationships, semisynthetic diversification, and molecular targets are integrated to highlight therapeutic opportunities and challenges. Particular emphasis is placed on covalent reactivity, selectivity, pharmacokinetic limitations, and strategies supporting the translational development of these promising natural‐product scaffolds.

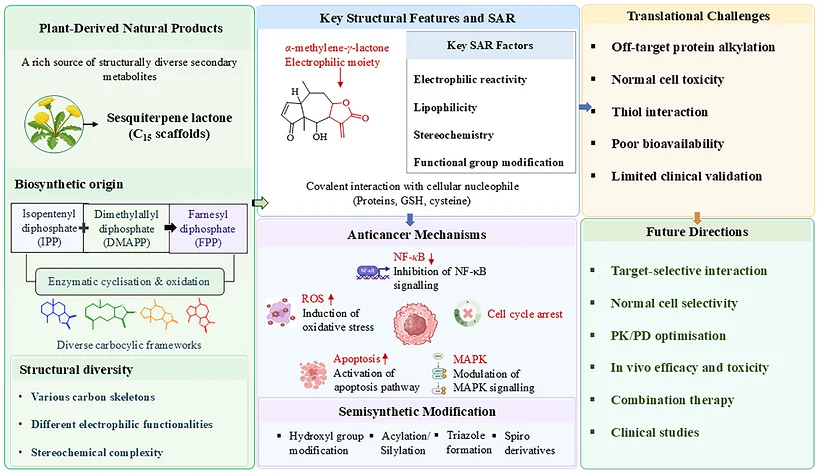

## 1 Introduction

Sesquiterpenes lactones (SLs) are an important class of secondary metabolites derived from plants and produced by diverse botanical families [1]. The highest structural diversity and largest number of SLs are found in the Asteraceae family, followed by Apiaceae, Lauraceae, Solanaceae, and Araceae [1, 2]. More than 8000 SL have been identified, reflecting their extensive chemical diversity [3]. In plants, SLs function primarily as chemical mediators involved in growth regulation and defence, protecting against herbivores, pathogens, and other predators [4]. Biosynthetically, SL arise by utilising universal isoprenoid precursors, isopentenyl pyrophosphate (IPP) and dimethylallyl diphosphate (DMAPP) [5, 6].

Structurally, SL compounds belong to the terpene class and are defined by a 15‐carbon (sesqui) backbone containing an *α*, *β*‐unsaturated carbonyl system and a characteristic *α*‐methylene‐*γ*‐lactone (olide) moiety [2, 4, 7]. The lactone ring is typically fused at the C‐6/C‐7 or C‐7/C‐8 position in either cis or trans configuration, with the trans orientation being predominant [8]. Based on variation in their carbocyclic frameworks, sesquiterpene lactones are classified into distinct structural groups, including guaianolides, pseudo‐guaianolides (5/7bicyclic fusion), germacranolides (10‐member ring), eudesmanolides, eremophilanolides, heliangolide (10‐membered ring with a 5‐membered fused lactone), and hyptocretenolides (5/7 bicyclic fusion) [9, 10, 11]. This pronounced structural diversity shows the wide range of biological activities attributed to SLs, including antimicrobial, cytotoxic, anti‐inflammatory, immunoregulatory action, and antifungal activity (Figure 1) [12, 13, 14].

**FIGURE 1:**
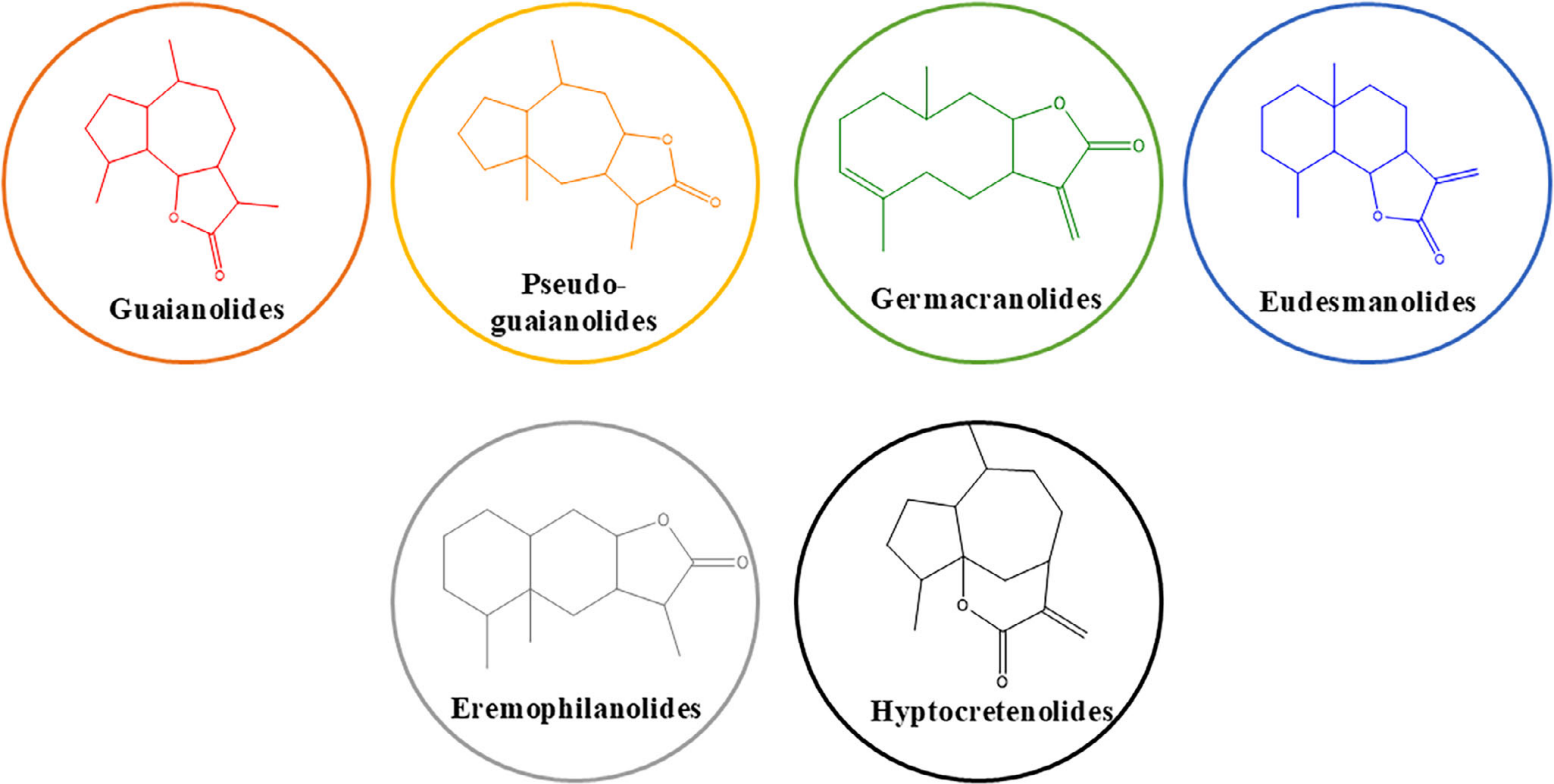
Structural classification of sesquiterpene lactones based on ring systems.

Over the past several decades, numerous review articles have highlighted the biological and pharmacological properties of SLs, including anticancer, anti‐inflammatory, antimicrobial, and other therapeutic activities [15, 16, 17, 18]. Several articles have also addressed specific aspects of SLs research, such as their biosynthetic pathways, structural diversity, molecular mechanisms of anticancer and anti‐inflammatory, and clinical relevance [19, 20, 21]. While existing articles have generally focused on individual aspects of SLs, such as chemistry and pharmacology or on selected biological activities. Consequently, an integrated view that connects the biosynthetic origin and chemical diversity of SLs with their structural activity relationship (SAR), anticancer mechanisms, chemical optimisation, and translational potential remains comparatively limited.

In this context, the present review provides an integrated framework that encompasses biosynthesis, chemical diversity, SAR, anticancer mechanisms, semisynthetic diversification, and clinical translation of SLs. Particularly focused on the structural features of the SLs scaffold and anticancer activity. To overcome limitations such as poor selectivity, stability, solubility, or pharmacokinetic properties of SLs by linking biosynthetic and structural considerations with mechanistic insights and semisynthetic approaches. This review aims to provide a progression from biosynthesis to potential clinical applications. Thus, rather than providing a general overview of the pharmacological activities of SLs.

### 1.1 Biosynthesis of Sesquiterpene Lactones and Derivatives

The biosynthesis of SLs is associated with the formation of the five‐carbon isoprenoid precursors, IPP and DMAPP [22]. These precursors are generated through the mevalonate (MVA) pathway in the cytosol and the 2‐C‐methyl‐D‐erythritol 4‐phosphate (MEP) pathway in plastids [23].

In the MVA pathway, condensation of two molecules of acetyl‐CoA yields acetoacetyl‐CoA, catalysed by acetyl‐CoA acetyltransferase (AACT) [24]. Subsequent condensation of acetoacetyl‐CoA with another molecule of acetyl‐CoA produces 3‐hydroxy‐3‐methylglutaryl‐CoA (HMG‐CoA), catalysed by hydroxymethylglutaryl‐CoA synthase (HMGS) [25]. HMG‐CoA is further reduced to MVA by 3‐hydroxy‐3‐methylglutaryl‐CoA reductase (HMGR) [22, 24, 25]. MVA is then converted into IPP through sequential phosphorylation and decarboxylation catalysed by mevalonate kinase (MK), phosphomevalonate kinase, and diphosphomevalonate decarboxylase (MVD). Subsequently, IPP isomerised to DMAPP by isopentenyl diphosphate isomerase (IDI) [26, 27].

The MEP pathway produces these precursors by condensing pyruvate and glyceraldehyde‐3‐phosphate (G3P) to form 1‐deoxy‐D‐xylulose‐5‐phosphate (DXP), catalysed by 1‐deoxy‐D‐xylulose‐5‐phosphate synthase (DXS) [28]. DXP is subsequently reduced to MEP by 1‐deoxy‐D‐xylulose‐5‐phosphate reductoisomerase (DXR) [29]. MEP is then converted into 4‐(cytidine 5′‐diphospho)‐2‐C‐methyl‐d‐erythritol (CD‐ME) by 2‐C‐methyl‐d‐erythritol 4‐phosphate cytidylyltransferase (MCT). Further phosphorylation by 4‐diphosphocytidyl‐2‐C‐methyl‐d‐erythritol kinase (CMK), cyclisation by 2‐C‐methyl‐d‐erythritol 2,4‐cyclodiphosphate synthase (MDS), and subsequent conversion by 4‐hydroxy‐3‐methylbut‐2‐enyldiphosphate synthase (HDS) lead to the formation of 1‐hydroxy‐2‐methyl‐2‐butenyl 4‐diphosphate (HMBPP). IPP and DMAPP are directly synthesised from HMBPP by 4‐hydroxy‐3‐methylbut‐2‐enyl diphosphate reductase (HDR) [28, 29].

The condensation of IPP and DMAPP yields farnesyl diphosphate (FPP), a key C15 intermediate that serves as the universal precursor for SLs biosynthesis [30]. Under the action of specific sesquiterpene synthases, FPP undergoes enzyme‐mediated cyclisation to form diverse sesquiterpene backbones [31, 32]. These intermediates are subsequently modified through NADPH‐dependent hydroxylation, NADP^+^‐dependent oxidation and intramolecular lactonisation involving a hydroxyl group at C‐6 or C‐8 and a carbonyl group at C‐12, leading to the characteristic *γ*‐lactone ring formation of SLs (Figure 2) [33, 34].

**FIGURE 2:**
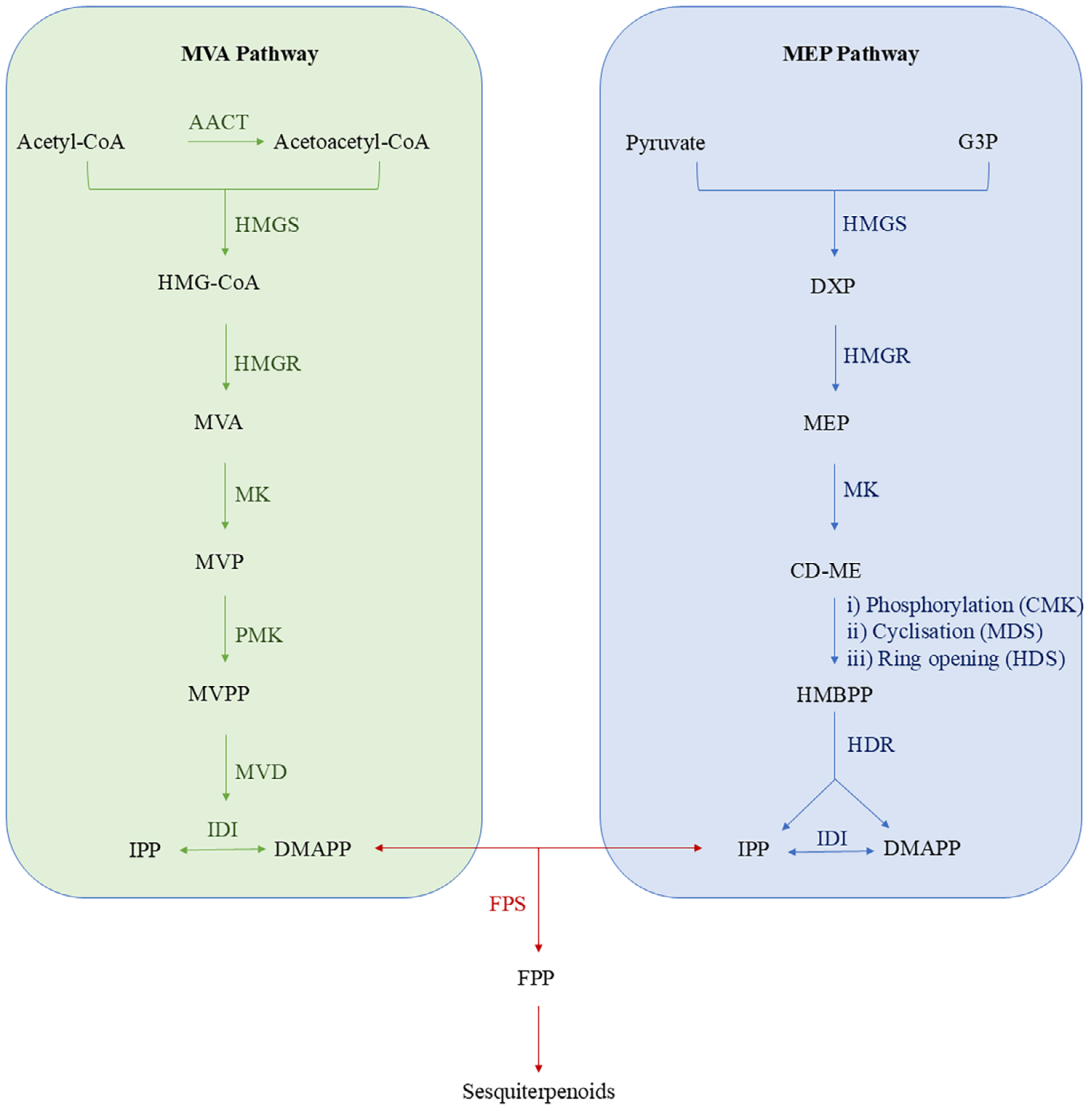
Biosynthetic routes to sesquiterpenes: mevalonate (MVA) pathway and the MEP pathway leading to IPP/DMAPP and FPP formation.

In chicory (*Cichorium intybus*) sprouts, (+)‐germacrene A is produced from FPP by (+)‐germacrene A synthase, which acts as a central biosynthetic intermediate for germacranolides, eudesmanolides and guaianolides [35]. Oxidation of germacrene A produces germacrene A acid (GAA), which is transformed into costunolide by costunolide synthases and subsequently converted into structurally complex SLs such as parthenolide (**9**) and O, O‐diacetylbritannilactone (**10**) [36, 37]. In *Helianthus annus*, GAA undergoes regioselective hydroxylation by the cytochrome p450 enzyme HaG8H (CYP71bL1) to form 8*β*‐hydroxy‐GAA, which lactonizes to yield inunolide, whereas further hydroxylation at C‐6 produces 6*α*,8*β*‐dihydroxy‐GAA, leading to eupatolide (**11**) formation [38]. In *Artemisia annua*, FPP is converted into amorpha‐4,11‐diene, which is sequentially oxidised to artemisinic alcohol and artemisinic aldehyde by amorpha‐4,11‐diene 12‐monooxygenase (CYP71AV1) [39, 40]. Subsequent reduction and oxidation steps yield dihydroartemisinic acid, which is ultimately converted into artemisinin (12) via a process that remains incompletely elucidated [36, 41].

### 1.2 Structural Features Influencing Sesquiterpene Lactones Bioactivity

The biological activity of SLs is primarily governed by the three key interrelated chemical attributes: electrophilic reactivity (Alkylating centres), lipophilicity, and molecular geometry [11]. Among these, the presence, number, accessibility, and spatial arrangement of electrophilic centres are particularly important for pharmacological activity. Reactive functionalities commonly associated with SL bioactivity include the *α*‐methylene‐ *γ*‐lactone, *α*‐methylene‐*δ*‐lactone, conjugated cyclopentenone, and other *α, β*‐ unsaturated or ester‐containing structural motifs [31]. SLs containing two electrophilic centres are generally described as bifunctional and exhibit greater biological potency than their monofunctional counterparts [42]. Thus, the biological effects of SLs can be rationalised by considering the relationships among structural functionality, electrophilic reactivity, protein target engagement, and downstream biological response (Figure 3).

**FIGURE 3:**
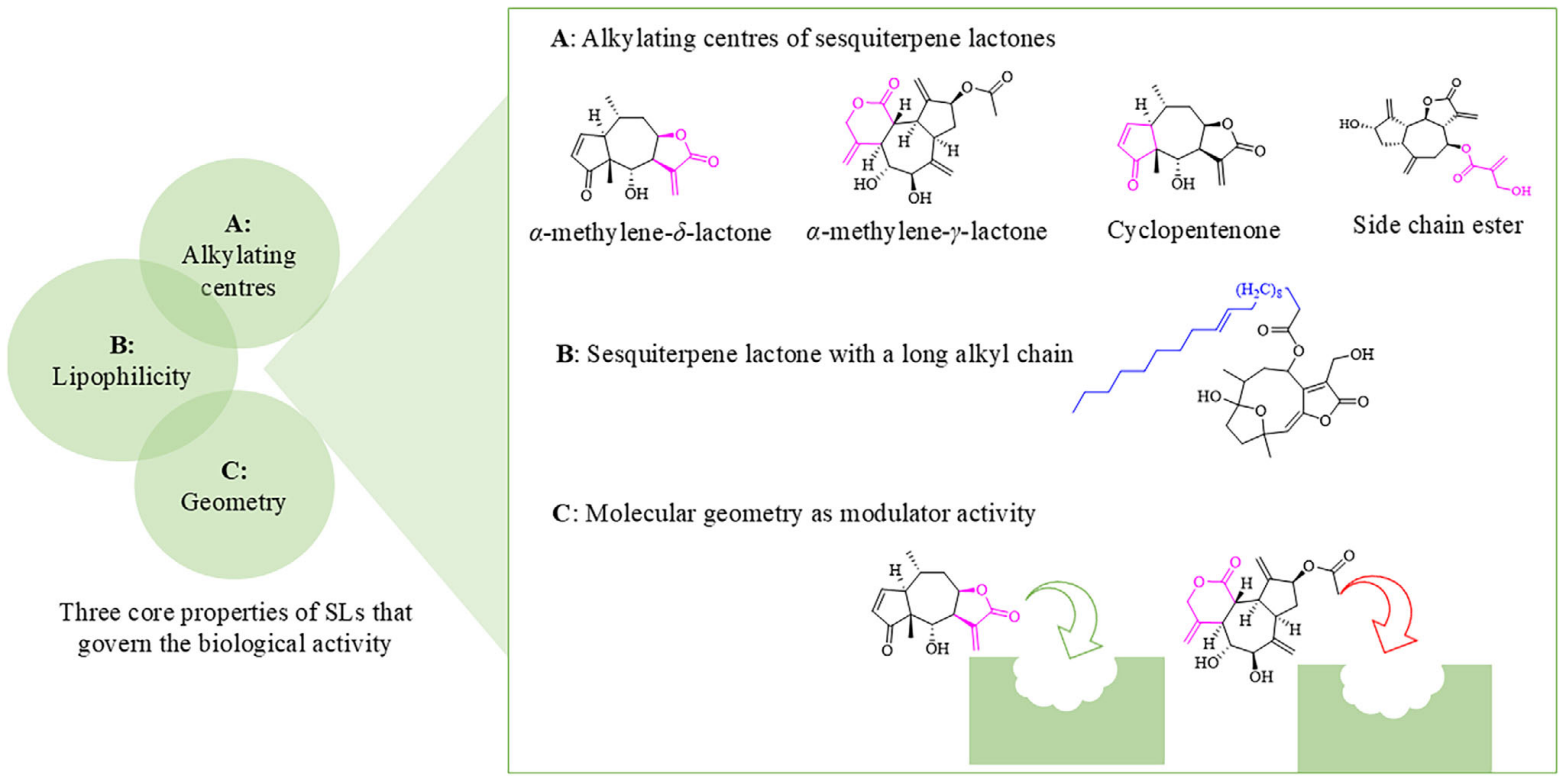
Structural determinants governing the biological activity of sesquiterpene lactones: (A) electrophilic alkylating centres (*α*‐methylene lactone and related reactive motifs), (B) lipophilicity modulated by alkyl side chains, and (C) molecular geometry.

### 1.3 Electrophilic Centres and Alkylating Activity

The *α*‐methylene‐*γ*‐lactone moiety represents one of the most important structural determinants of SLs activity that is present in either exocyclic or endocyclic configurations. This *α*,*β*‐ unsaturated lactone functions as a Michael acceptor, allowing covalent interaction with nucleophilic thiol groups, particularly those of cysteine residues in cellular proteins [43, 44]. Such covalent interaction can alter the activity of redox‐sensitive proteins and signalling pathways, ultimately affecting cell survival, proliferation, inflammation, and apoptosis.

Helenalin (**1**), mexicanin‐I (**2**), and parthenin (**3**) are highly active SLs containing multiple electrophilic functionalities. Their enhanced cytotoxicity has been associated with the presence of bifunctional electrophilic systems, including the 14,15*α*‐methylene‐*γ*‐lactone, *α*‐methylene‐*δ*‐lactone, and conjugated cyclopentenone moieties [45, 46]. In contrast, reduction or removal of the double bond, as observed in 11*α*, 13‐dihydroarnifolin and 11*α*, 13‐dihydrochamissonolide, markedly decreases cytotoxicity, demonstrating the importance of an intact Michael acceptor for biological activity.

### 1.4 Number and Position of Electrophilic Centres

The number and position of the electrophilic centres further influence target engagement and biological potency [43]. Helenalin (**1**) exhibits high reactivity towards the thiol‐containing proteins, including the p65 subunits of NF‐*κ*B, providing a mechanistic basis for its anti‐inflammatory and cytotoxic effects [47]. The presence of two electrophilic centres may therefore permit interaction with multiple nucleophilic sites or enhance the probability of covalent target engagement.

Helenalin (**1**) and mexacanins I (**2**) derivatives possessing an ester substituted at C‐6 together with an exocyclic double bond at C‐1 show enhanced cytotoxicity, which has been attributed to their bifunctional electrophilic character [46]. Conversely, the 11*α*,13‐dihydrohelenalins derivative (**7**), containing only one effective alkylating centre, exhibits reduced activity compared to bifunctional analogues [46]. These indicate that electrophile multiplicity and spatial arrangement can determine the extent of protein modification and biological response.

### 1.5 Contribution of the Cyclopentenone Moiety

The cyclopentenone moiety provides an additional electrophilic centre that can balance the reactivity of the *α*‐methylene lactone. Its conjugated carbonyl‐alkene system can act as a Michael acceptor, enhancing interactions with nucleophilic residues in regulatory proteins. Modification or saturation of this electrophilic system reduces biological activity, emphasising that preserving the conjugated double bond is important for maintaining reactivity. In particular, changes affecting the cyclopentenone electrophile have been associated with reduced NF‐*κ*B binding and biological response [45, 46]. Similarly, structural modification of the cyclopentenone double bond can reduce NF‐*κ*B interaction and alter downstream effects, including telomerase inhibition [45, 46]. Therefore, the combined presence of the *α*‐methylene lactone and cyclopentenone electrophile generates a bifunctional pharmacophore that produces stronger biological responses than a single electrophile centre.

Lactone type and degree of unsaturation also contribute to activity. Conversion of the *α*‐methylene‐*γ*‐lactone into a non‐electrophilic *γ*‐lactone that reduces cytotoxicity. For example, 11*β*,13‐dihydrocostunolide exhibits cytotoxicity that is ∼40‐fold lower than that of the corresponding *α*‐methylene‐*γ*‐lactone‐containing analogue, costunolide (**6**) [48]. This comparison provides strong evidence that the electrophilic exocyclic methylene group, rather than the lactone ring alone, is a major contributor to cytotoxic potency.

### 1.6 Hydroxylation and Stereochemistry

Hydroxylation and stereochemistry further regulate SL activity by altering electrophilic accessibility, hydrogen bonding capacity, polarity, and three‐dimensional target recognition. An increase in hydroxyl substitution on *α*‐methylene‐*γ*‐lactone‐containing compounds generally decreases cytotoxicity due to increased polarity and steric effects that influence membrane permeability and access to the electron centre [48]. Importantly, the biological consequences of hydroxylation are strongly dependent on its position and configuration. For example, 3‐epizaluzanin C (**4**), possessing an *α*‐oriented hydroxyl group at C‐3, exhibits greater cytotoxicity than its *β*‐hydroxyl epimer (Zaluzanin C), demonstrating that stereochemical configuration can determine the orientation and accessibility of functional groups involved in the target recognition [48].

Molecular geometry additionally influences the spatial presentation of electrophilic groups and other pharmacophoric features to biological targets. Although molecular geometry may have a comparatively smaller contribution than the electrophilic reactivity of some SL activities, stereochemical orientation and conformational constraints can influence target recognition and biological response. The exocyclic methylene group and *α*,*β*‐unsaturated cyclopentenone system have been particularly implicated in anti‐inflammatory activity, While three‐ dimensional arrangement of the SL scaffold can determine the accessibility of the reactive functionality [49]. Beyond the major electrophilic pharmacophore, individual structural motifs may confer specialised biological activities. Cynaropicrin (**8**) exhibits smooth muscle inhibitory activity associated with its *γ*‐butyrolactone [3, 50] (Table 1).

**TABLE 1:** Summary of the major structural determinants governing the structure‐activity relationships (SAR) of sesquiterpene lactones, including lactone electrophilicity, additional reactive centres, hydroxylation, esterification, lipophilicity, and stereochemistry, and their effects on target engagement and biological activity.

Structural feature	Reactivity/physicochemical effect	Target	Biological outcome	SAR interpretation	Ref.
α‐Methylene‐γ‐lactone	Strong Michael acceptor; covalent reaction with cysteine thiols	NF‐κB‐associated proteins and other nucleophilic cellular proteins	Cytotoxicity, apoptosis, and anti‐inflammatory activity	Loss/reduction of electrophilicity generally decreases activity	[53]
α‐Methylene‐δ‐lactone	Electrophilic Michael acceptor	Nucleophilic protein residues	Cytotoxic and other pharmacological effects	Contributes to electrophilic/bifunctional character	[54]
Cyclopentenone	α, β‐Unsaturated electrophilic centre	Redox‐sensitive and signalling proteins	Cytotoxicity, anti‐inflammatory effects	An additional electrophilic centre can enhance potency	[55]
Two electrophilic centres	Increased potential for covalent target engagement	Multiple nucleophilic protein sites	Enhanced cytotoxic/pharmacological activity	Bifunctional SLs often show greater potency than monofunctional analogues	[56]
Hydroxylation	Increases polarity/H‐bonding; may alter electrophile accessibility	Target recognition and cellular uptake	Can increase or decrease activity depending on position	Position and configuration are critical	[48]
Esterification	Increases lipophilicity and masks free hydroxyl groups	Cellular uptake and molecular recognition	Changes cytotoxic potency and pharmacokinetics	Can improve permeability, but may reduce activity when free OH is required	[48]
Lipophilicity	Influences membrane permeability, distribution and solubility	Intracellular target exposure	Modulates cytotoxicity and bioavailability	Optimal lipophilicity is preferable; excessive lipophilicity may impair in vivo behaviour	[45]
Stereochemistry	Controls 3D orientation/accessibility of pharmacophores	Stereoselective target recognition	Changes potency and selectivity	The α/β configuration can markedly alter activity	[48]
γ‐Lactone without electrophilic methylene	Reduced electrophilic reactivity	Reduced covalent target engagement	Markedly reduced cytotoxicity	Demonstrates the importance of α‐methylene functionality	[45, 46]

### 1.7 Adverse and Genotoxic Effects Associated With Electrophilic Reactivity

Several SLs have adverse effects beyond their pharmacological effects; for example, SLs containing an *α*‐methylene‐*γ*‐lactone covalently react with nucleophilic cysteine residues in skin proteins (carrier‐hapten complex), are modified, and act as antigens capable of inducing a cell‐mediated type IV hypersensitivity reaction [43, 51]. *α*‐methylene‐*γ*‐lactone is highly photoreactive towards thymine/thymidine to form photoadducts, which can cause chronic actinic dermatitis [43, 51]. Helenalin (**1**) and hymenovin are reported for the first time as lethal mutagens in *Bacillus subtilis* and *Bacillus thuringiensis* by alkylating two guanines and causing DNA cross‐linkage (Norman, Johnson, Mollenhauer and Meola [51, 52]. Many SLs show genotoxicity properties by performing micronucleus (MN), sister chromatid exchange (SCE)and chromosomal aberration (CA) assays, such as centratherin, which increases the SCE rate in human peripheral blood lymphocytes, and glaucolide B, which causes clastogenic effects by inducing structural CA in vitro but not in vivo (mouse bone marrow assay) [51].

Collectively, the SAR of SLs demonstrates that biological activity results from the integrated contribution of electrophilic reactivity, number and positioning of reactive centres, lactone unsaturation, hydroxylation, esterification, lipophilicity, and stereochemical configuration. The *α*‐methylene‐*γ*‐lactone and cyclopentenone functionalities govern covalent reactivity with nucleophilic protein residues, whereas hydroxylation, esterification, and lipophilicity govern accessibility and membrane permeability. These structural factors ultimately influence interaction with molecular targets such as NF‐*κ*B‐associated proteins and other cysteine‐containing cellular regulators, resulting in downstream effects including apoptosis, inhibition of proliferation, modulation of inflammation, and alteration of redox signalling.

## 2 Article Selection Criteria

A literature search was conducted in PubMed, Scopus, Science Direct, and Google Scholar to identify publications on SLs, their Biosynthesis, SAR, and anticancer potential. The search included keywords such as “sesquiterpene lactones”, “biosynthesis structure‐activity relationship”, “semisynthetic modification”, “anti‐cancer activity”, and “anti‐inflammatory activity” of SLs. Relevant literature published between 1994 and 2026 was considered. Relevant peer‐reviewed original research and review articles were included based on keywords searched, and duplicate, unrelated, and insufficiently relevant publications were excluded. The reference lists of the selected articles were also screened to identify additional relevant studies on SLs.

## 3 Anticancer Activity of Sesquiterpene Lactones

Sesquiterpene lactones are biologically significant plant‐derived natural products that have attracted considerable scientific interest owing to their pronounced anticancer activity [57]. The anticancer effects of SLs are primarily mediated by electrophilic *α*,*β* unsaturated carbonyl functionalities, particularly the *α*‐methylene‐*γ*‐lactone and cyclopentenone moieties, which enable covalent interactions with key cellular targets (Table 2) (Figure 4) [3, 58].

**TABLE 2:** Comprehensive overview of cytotoxic sesquiterpene lactones: source, cell line activity, and molecular mechanisms.

Sr.no.	Compound	Class	Biological source	Cell line	Reported Concentration	IC_50_ (µM)	Response/endpoint	Mechanism	Ref.
1.	Helenalin (**1**)	Guaianolide	*Viguiera buddleiaeformis*	EN‐2	2.4 ± 0.4 µM	2.4 ± 0.4	IC_50_ reported	—	[46]
			*Arnica chamissonis*	ATCC CCL 17	0.64 ± 0.09 µM	0.64 ± 0.09	IC_50_ reported	Covalently binds to sulfhydryl groups of essential proteins via Michael‐type addition	[59]
2.	2*β*‐(*S)*‐Glutathionyl‐2,3‐dihydrohelenalin	—	—	ATCC CCL 17	1.2 ± 0.01 µM	1.2 ± 0.01	IC_50_ reported	Covalently binds to sulfhydryl groups of essential proteins via Michael‐type addition	[59]
3.	11*α*,13‐tetrahydrohelenalin acetate	Guaianolide	*Arnica chamissonis*	ATCC CCL 17	6.3 ± 0.31 µM	6.3 ± 0.31	IC_50_ reported	Covalently binding to sulfhydryl groups of essential proteins via Michael‐type addition	[59]
4.	2*β*‐(*S*)‐Glutathionyl‐2,3,11*α*,13‐tetrahydrohelenalin acetate	—	—	ATCC CCL 17	6.5 ± 1 µM	6.5 ± 1	IC_50_ reported		
5.	11R,13‐dihydrohelenalin (**7**)	Guaianolide	*Viguiera buddleiaeformis*	EN‐2	128 ± 17 µM	128 ± 17	IC_50_ reported	—	[46]
6.	Mexicanin I (**2**)	Guaianolide	*Viguiera buddleiaeformis*	EN‐2	2.7 ± 0.67 µM	2.7 ± 0.67	IC_50_ reported	—	[46]
7.	Leptocarpin (**13**)	Guaianolide	*Leptocarpa rivularis*	HT‐29	2.0 ± 0.8 µM	2.0 ± 0.8	IC_50_ reported	NF‐*κ*B pathway inhibition, Chromatin condensation, nuclear fragmentation, and cytochrome C release in the cytosol. Increase caspase‐3 activity.	[60]
				PC‐3	4.5 ± 0.3 µM	4.5 ± 0.3			
				DU‐145	3.8 ± 0.2 µM	3.8 ± 0.2			
				MCF7	3.1 ± 0.3 µM	3.1 ± 0.3			
				MDA‐MB‐231	6.4 ± 0.5 µM	6.4 ± 0.5			
				CCD 841CoN	5.2 ± 0.3 µM	5.2 ± 0.3			
				HDF (NCC)	18.7 ± 3.5 µM	18.7 ± 3.5			
8.	Bigelovin (**14**)	Guaianolide	*Inula helianthus aquatica*	HepG2 SMMC‐ 7721 HSC‐LX2 (NCC)	2.5 to 10 µM	—	—	Activation of Caspase‐9,3 and PARP cleavage. Reduction of p62. LC3BII upregulation of protein. Overexpression of p‐mTOR. Elevation in the production of ROS	[61]
9.	Lappalone (**26**)	Guaianolide	*Saussaurea lappa*	HeLa	3 µg/mL	—	—	—	[48]
				OVCAR‐3	3 µg/mL				
				HepG2	>100 ug/mL				
10.	Lappadilactone (**24**)	Guaianolide	*S. lappa*	HeLa	1.8 µg/mL	—	—	—	[48]
				OVCAR‐3	2.4 µg/mL				
				HepG2	2.5 µg/mL				
11.	Dehydrocostus lactone (**25**)	Guaianolide	*S. lappa*	HeLa	3.5 µg/mL	—	—	—	[48]
				OVCAR‐3	3.5 µg/mL				
				HepG2	2.5 µg/mL				
12.	Costunolide (**6**)	Germacranolides	*S. lappa*	HeLa	2.0 µg/mL	—	—	—	[48]
				OVCAR‐3	1.6 µg/mL				
				HepG2	2.0 µg/mL				
13.	9‐acetoxycostunolide (**38**)	Germacranolide	*Cyathocline purpurea*	L1210	0.89 ± 0.06 µg/mL	3.06 ± 0.21^a^	IC_50_ reported	Inhibit mitosis and reduce thymidine uptake (L1210); Caspase 3 activation (L1210)	[62]
				CCRF‐CEM	0.41 ± 0.03 µg/mL	1.41 ± 0.10^a^			
				KB	0.25 ± 0.02 µg/mL	0.86 ± 0.07^a^			
				LS174T	1.28 ± 0.05 µg/mL	4.41 ± 0.17^a^			
				MCF‐7	0.63 ± 0.0.7µg/mL	2.17 ± 0.24^a^			
14.	Parthenolide (**9)**	Germacranolides	*Tanacetum parthenium*	SH‐J1	5‐10 µM	—	—	Delayed cell cycle by arresting G2/M phase. DNA fragmentation (Induces apoptosis). Increases generation ROS by 3‐fold. Overexpression of the GADD153 gene	[63]
15.	9‐Acetoxyparthenolide (**39**)	Germacranolide	*C. purpurea*	L1210	0.59 ± 0.05 µg/mL	1.93 ± 0.16^a^	IC_50_ reported	Inhibit mitosis and reduce thymidine uptake (L1210). Caspase 3 activation (L1210)	[62]
				CCRF‐CEM	0.49 ± 0.07 µg/mL	1.60 ± 0.23^a^			
				KB	0.29 ± 0.01 µg/mL	0.95 ± 0.03^a^			
				LS174T	1.08 ± 0.09 µg/mL	3.53 ± 0.29^a^			
				MCF‐7	0.50 ± 0.03 µg/mL	1.63 ± 0.10^a^			
16.	Iso‐seco‐tanapartholide (TNP) (**19**)	Germacranolide	*Achillea falcata*	HCT‐116 DLD‐1	10 µg/mL 10 µg/mL	—	Cell viability decreased by 20%	ROS‐dependent apoptosis. Disruption of the mitochondrial membrane potential	[64]
17.	SalA: TNP (combination therapy)	—	*Centaurea ainetensis;* *Achillea falcata*	HCT‐116 DLD‐1	Sal A: 3 µg/mL: TNP: 5 µg/mL	—	Cell viability decreases by 80%	ROS‐dependent apoptosis (25 times more). Disruption of the mitochondrial membrane potential. MAPKs activation mediated cell death (p‐ERK, p‐p38, p‐JNK.	[64]
18.	Saligraviolide (Sal A) (**18**)	Germacranolide	*C. ainetensis*	HCT‐116 DLD‐1	116 µg/mL 8 µg/mL	—	Cell viability decreases by 20%	ROS‐dependent apoptosis. Disruption of the mitochondrial membrane potential	[64]
19.	Alantolactone (**15**)	Eudesmanolide	*I. helenium*	SH‐SY5Y	20.24 ± 2.11 µM	20.24 ± 2.11	IC_50_ reported	Inhibiting the NF‐*κ*B and COX‐2 signalling pathway. Cyt c/caspase‐dependent pathway activation by suppressing COX‐2 expression Downregulation of MMP‐2 and MMP‐9 proteins.	[65]
				U251	16.33 ± 1.93 µM	16.33 ± 1.93			
				U118	29.16 ± 2.84 µM	29.16 ± 2.84			
				U87	24.06 ± 2.38 µM	24.06 ± 2.38			
20.	Isoalantolactone (**23)**	Eudesmanolide	*A. afra*	HeLa EN‐2	8.15 ± 1.16 µM 62 ± 7.4 µM	8.15 ± 1.16 62 ± 7.4	IC_50_ reported	—	[66]
21.	8‐ *α*‐erucyl‐1*α*‐hydroxyl hirustinolide (**20**)	Hirustinolide	*C. cinereum (L.)*	786‐O cell K‐562	12.5 µg/mL 12.8 µg/mL	—	—	Generation ROS. Inducing S phase arrest (affecting DNA replication)	[67]
22.	1*α*‐hydroxyhirustinolide‐13‐O‐acetate (**21**)	Hirustinolide	*C. cinereum (L.)*	786‐O cell K‐562 MCF‐7	17.7 µg/mL 12.2 µg/mL 17.6 µg/mL	—	—	—	[67]
23.	8‐ *α*‐erucyl‐1*α*‐hydroxyl hirustinolide 1R,5,8R‐trihydroxygermacre‐4(15).9(Z),11(13)‐trien‐6R,12‐olide (**27)**	Class: Hirustinolide; subclass: Germacranolide	*C. cinereum (L.)*	786‐O K‐562	12.5 µg/mL 12.8 µg/mL	—	—	Generation ROS. Inducing S phase arrest (affecting DNA replication).	[67]
24.	Millerenolide (**30)**	Guaianolide	*Viguiera sykavatica*	B16/BL6	43.0 µg/mL	115	IC_50_ reported‐	—	[68]
				A549	15.0 µg/mL	40			
				NIH‐3T3 (NCC)	20.0 µg/mL	54			
				NIH‐3T3/HER2 (NCC)	6.0 µg/mL	16			
25.	Thieleanin (**31)**	Guaianolide	*Decachaeta thieleana*	B16/BL6	>50 µg/mL	203	IC_50_ reported	—	[68]
				A549	8.0 µg/mL	32			
				NIH‐3T3 (NCC)	41.0 µg/mL	166			
				NIH‐3T3/HER2 (NCC)	7.0 µg/mL	28			
26.	1R,5,8R‐trihydroxygermacra 4(15),9(Z),11(13)‐trien‐6R,12‐olide (**27**)	Germacrane	*G. gomerae*	HL‐60	16.0 ± 3.6 µM	16.0 ± 3.6	IC_50_ reported	—	[46]
				U‐937	10 ± 0.3 µM	10 ± 0.3			
				SK‐MEL‐1	19.5 ± 3.7 µM	19.5 ± 3.7			
				A549	56.6 ± 15.1 µM	56.6 ± 15.1			
27.	Dentantin A (**28)**	Germacranolide	*G. frutigosum*	HL‐60	9.4 ± 0.7 µM	9.4 ± 0.7	IC_50_ reported	—	[69]
				U‐937	9.2 ± 1.1 µM	9.2 ± 1.1			
				SK‐MEL‐1	14.3 ± 3.2 µM	14.3 ± 3.2			
				A549	72.4 ± 13.7 µM	72.4 ± 13.7			
28.	1*β*,4R,6R‐trihydroxyeudesm‐11‐en‐8R,12‐olide	Eudesmanolide	*G. frutigosum*	HL‐60	13.3 ± 2.5 µM	13.3 ± 2.5	IC_50_ reported	—	[69]
				U‐937	10.4 ± 1.0 µM	10.4 ± 1.0			
				SK‐MEL‐1	27.9 ± 4.1 µM	27.9 ± 4.1			
				A549	>100 µM	>100			
29.	Chamissonolide (**33**)	Guaianolide	—	EN‐2	106 ± 17 µM	106 ± 17	IC_50_ reported	—	[46]
			*A. chamissonis*	ATCC CCL 17	2.3 ± 0.15 µM	2.3 ± 0.15	IC_50_ reported	Covalently binding to sulfhydryl groups of essential proteins via Michael‐type addition	[59]
30.	13‐S‐Glutathionyldihydrochamissonolide	—	—	ATCC CCL 17	> 15 µM	> 15	IC_50_ reported	Covalently binding to sulfhydryl groups of essential proteins via Michael‐type addition	[59]
31.	Arglabin (**34**)	guaianolide	*A. glabella*	SCC‐4	10 µM	—	—	Induction of mitochondrial‐mediated apoptosis. Reduces the migration of cancer cells. mTOR/PI3K/Akt pathway invasion and inhibition.	[70]
32.	Santamarin (**37**)	Germacranolide	*C. purpurea*	L1210	0.41 ± 0.03 µg/mL	1.65 ± 0.12^a^	IC_50_ reported	Inhibit mitosis and reduce thymidine uptake (L1210)Caspase 3 activation (L1210)	[62]
				CCRF‐CEM	0.59 ± 0.12 µg/mL	2.38 ± 0.48^a^			
				KB	0.16 ± 0.03 µg/mL	0.64 ±0.12^a^			
				LS174T	0.92 ± 0.01 µg/mL	3.71 ± 0.04^a^			
				MCF‐7	0.53 ± 0.10µg/mL	2.13 ± 0.40^a^			

Abbreviation: NCC, Normal‐cell comparator.

a (‐) Data not available in the referred article, (^a^) Values in µM are calculated based on the reported concentration in µg/mL.

FIGURE 4Structures of naturally occurring sesquiterpene lactones investigated for biological activity.

**Figure:**
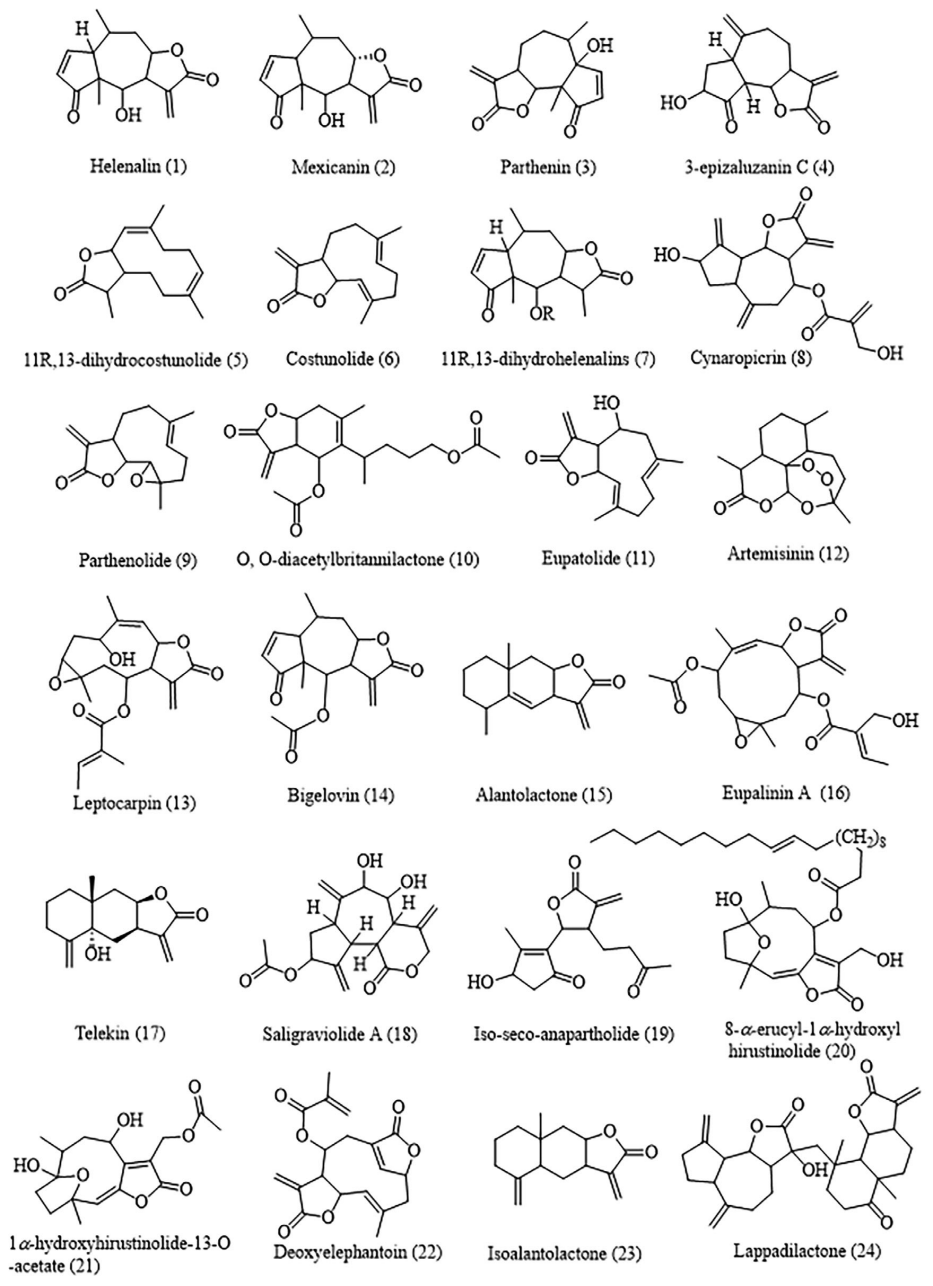

**Figure:**
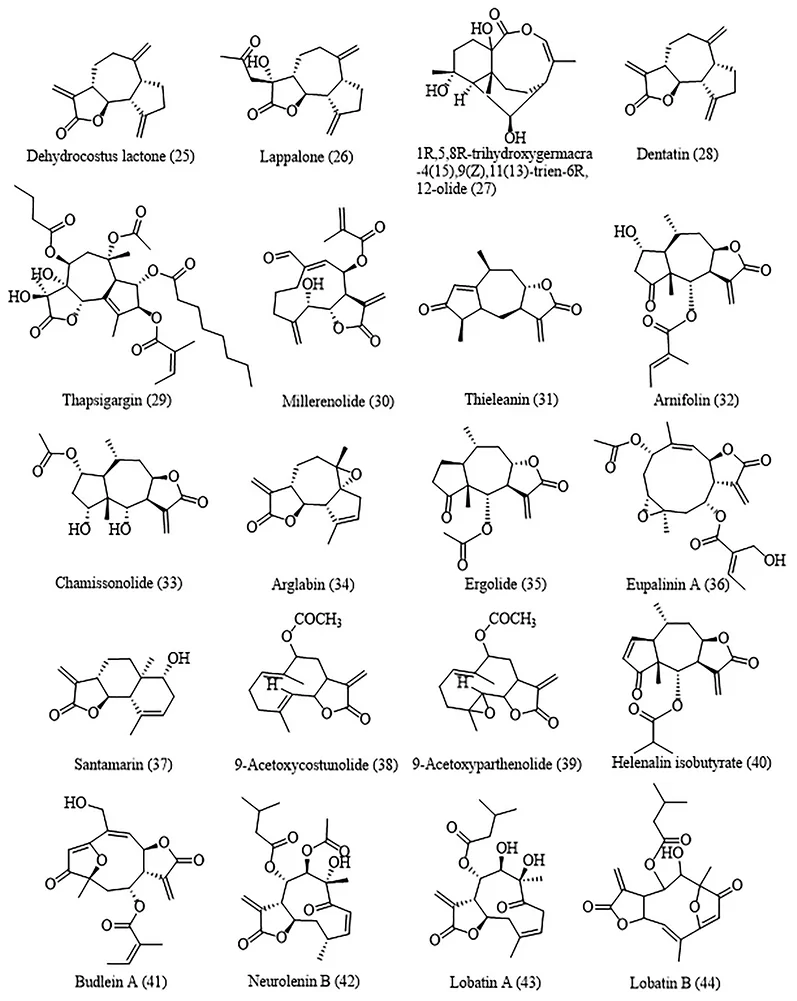

### 3.1 Nuclear Transcription Factor‐*κ*B (NF‐*κ*B) Signalling Pathway

The anticancer activity of SLs is largely mediated by inhibition of the NF‐*κ*B signalling pathway, which is essential for cell proliferation, metastasis, resistance to apoptosis, and resistance to chemotherapy and radiotherapy [71, 72]. NF‐*κ*B is a crucial transcription factor regulating genes associated with inflammation, immune responses, cell cycle progression, and cell survival [73]. Structurally, NF‐*κ*B exists as a heterodimer primarily composed of p50 and p65 subunits, which are retained in the cytoplasm by the inhibitory protein I*κ*B [74, 75]. Upon cellular stimulation, I*κ*B kinase *β* (IKK*β*) phosphorylates I*κ*B, triggering its proteasomal degradation and permitting NF‐*κ*B to translocate to the nucleus, thereby activating the transcription of desired genes [76]. NF‐*κ*B promotes the expression of anti‐apoptotic proteins, including membrane proteins of the Bcl‐2 family (e.g., Bcl‐2 and Bcl‐XL), inhibitors of apoptosis proteins, and antioxidant enzymes such as superoxide dismutase [77]. Inhibition of NF‐*κ*B signalling, including suppression of tumour necrosis factor (TNF)‐*α*‐induced activation, therefore represents a key molecular strategy for inducing apoptosis in cancer cells (Figure 5) [78].

**FIGURE 5:**
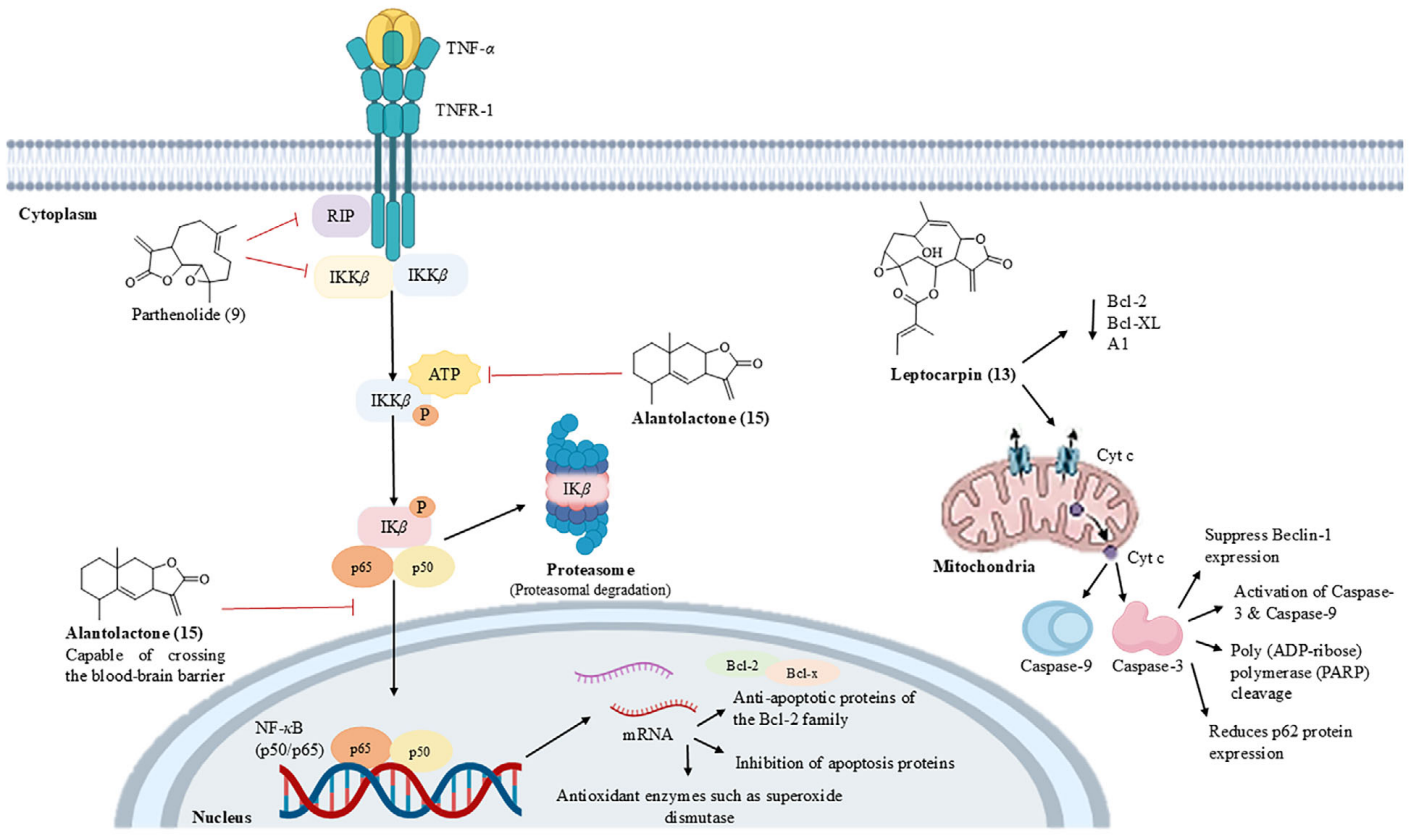
Inhibition of the NF‐κB signalling pathway by sesquiterpene lactones: molecular targets of parthenolide and alantolactone. (TNF‐α, tumour necrosis factor‐alpha; TNFR1, tumour necrosis factor receptor 1; RIP, receptor interaction protein; NF‐κB, nuclear transcription factor‐κB, IKKβ, IκB kinase β).

Several SLs have been shown to exert potent anticancer effects by modulating this pathway. Leptocarpin (**13**) induces apoptosis by inhibiting the NF‐*κ*B signalling pathway, resulting in the downregulation of the anti‐apoptotic proteins Bcl‐2 and Bcl‐XL and the activation of the mitochondrial apoptotic pathway through increased mitochondrial membrane permeability and cytochrome c release [60, 79]. Leptocarpin (**13**) exhibits dose‐dependent cytotoxicity in HT‐29, OC‐3, DU‐145, MCF7, and MDA‐MB‐231 cell lines, with IC_50_ values ranging from 2.0 to 6.4 µM. In comparison, it shows comparatively lower toxicity toward human dermal fibroblasts (IC_50_ = 18.7 µM) [60]. Bigelovin (**14**) suppresses NF‐*κ*B‐associated survival signalling by decreasing beclin‐1 expression, activating Caspase‐9 and Caspase‐3, inducing Poly (ADP‐ribose) polymerase (PARP) cleavage, and reducing p62 protein expression in HepG2 and SMMC‐7721 cells at concentrations of 2.5–10 µM [61].

Parthenolide (PH) (**9**) inhibit NF‐*κ*B activation by preventing the recruitment of I*κ*B kinase *α* (IKK*α*) and IKK*β* to the tumour necrosis factor receptor 1 (TNFR1) and disrupting the interaction between receptor‐interacting protein (RIP) and TNFR1, thereby impairing downstream signalling [78]. Alantolactone (**15**) suppresses glioblastoma cells' proliferation by inhibiting COX‐2 and IKK*β* activity at the adenine triphosphate (ATP)‐binding site and blocking nuclear translocation of the p65/p50 complex; notably, alantolactone is capable of crossing the blood‐brain barrier, enhancing its therapeutic relevance [69].

### 3.2 Role of Oxidative Stress in Sesquiterpene Lactone‐Mediated Anticancer Activity

Reactive oxygen species (ROS) comprise oxygen‐derived free radicals, superoxide anion, alkoxyl, hydroxyl, peroxyl radicals, as well as non‐radical species such as hydrogen peroxide (H_2_O_2_), singlet oxygen, alkyl peroxides, which are generated by the mitochondrial electron transport chain or oxidoreductase enzymes such as xanthine oxidase, lipoxygenase, cyclooxygenases (COXs), and NADPH oxidases (NOXs) [80, 81, 82]. Among these, hydroxyl radicals and singlet oxygen directly damage DNA, whereas protein thiol (–SH) groups, particularly those of cysteine residues, are readily oxidised by ROS, leading to functional alterations in cellular proteins, causing molecular damage including point mutations, single‐strand breaks, miscoding, abnormal gene amplification, and oncogene activation, ultimately triggering apoptotic cell death [53, 80, 83, 84]. At the physiological level, ROS function as the redox signalling molecules that regulate gene expression, metabolism, cell differentiation, proliferation, and apoptosis. However, excessive ROS generation disrupts redox homeostasis and induces cytotoxicity, ultimately leading to cell death [85].

Peroxiredoxin enzymes play a crucial role in cellular defence by controlling oxidative stress, in which Cys173 of PRDX1 and Cys172 of PRDX2 are essential for the peroxidase catalytic activity [86]. Ainsliadimer A directly target the antioxidant peroxiredoxin enzymes, such as Cys173 of PRDX1 and Cys172 of PRDX2, reduces their peroxidase activity, induces G2/M cell cycle arrest and apoptosis in colorectal cancer cells [86]. Several sesquiterpene lactones induce anticancer effects through ROS‐mediated mechanisms. Parthenolide (**9**) and costunolide (**6**) generate oxidative stress in MDA‐MB‐231 cells in a time‐dependent manner, with significant ROS production observed within the first three hours of treatment [87, 88, 89]. This effect is mediated by the activation of NADPH oxidase (NOX), resulting in increased superoxide anion generation, activation of c‐Jun N‐terminal kinase (JNK) by interacting with redox‐sensitive proteins like glutaredoxin and thioredoxin, inhibition of NF‐*κ*B signalling, and depletion of intracellular glutathione (GSH) [17, 90, 91]. Similarly, Other sesquiterpene lactones, including helenalin (**1)**, alantolactone (**15**), eupalinin A (**16**), and telekin (**17**), induce apoptosis in various cancer cell lines through enhanced ROS generation [85, 92, 93, 94].

Apart from their direct interaction with cellular proteins, the toxicity of SLs can be substantially influenced by the availability of intracellular thiols, particularly cysteine and GSH. Intracellular cysteine and GSH may protect cells against SL‐induced toxicity by competing with cellular proteins for reaction at electrophilic sites on SLs [59]. The reactivity of the two major electrophilic centres of SLs towards cysteine. The *α*‐methylene‐*γ*‐lactone moiety reacts extremely rapidly with cysteine (t_1/2_ <5 min), whereas the cyclopentenone moiety reacts more slowly (t_1/2_ ∼1000 min). Consequently, cysteine can preferentially and rapidly trap SLs containing an *α*‐methylene‐*γ*‐lactone, thereby reducing their availability for covalent interaction with cellular protein targets. Indeed, at a cysteine‐to‐SL ratio of 50:1, cysteine completely abolished the cytotoxicity of chamissonolide **(33)** and helenalin **(1)**, whereas it protected against 11*α*,13‐dihydrohelenalin acetate [59].

The interaction of SLs with GSH is more complex and strongly dependent on pH and the nature of the electrophilic centre [95]. Reaction between GSH and SLs is strongly pH‐dependent and proceeds more rapidly under physiological conditions (pH = 7‐8) than under acidic conditions. Notably, under these conditions, the cyclopentenone site reacts ∼10 times faster with GSH than the *α*‐methylene‐*γ*‐lactone site. Furthermore, GSH conjugation may have different consequences depending on the reactive site. Reaction of GSH with the cyclopentenone moiety is reversible, allowing partial regeneration of the free SL and thereby maintaining the potential for biological activity and toxicity. In contrast, conjugation at the *α*‐methylene‐*γ*‐lactone site is irreversible under physiological conditions [95, 96]. Thus, intracellular thiol availability may be an important factor in the balance among SL‐induced protein modification, pharmacological activity, and toxicity.

This protective role of thiol conjugation, glutathione adducts generally exhibited reduced cytotoxicity compared with their corresponding SLs. For example, 2*β*‐*S*‐Glutathionyl‐2,3‐dihydrohelenalin exhibited an IC_50_ of 1.2 ± 0.01 µM against the KB cells (ATCC CCL17), compared with 0.64 ± 0.09 µM for helenalin (**1**). Similarly, 2*β*‐*S*‐Glutathionyl‐2,3,11*α*,13‐tetrahydrohelenalin acetate exhibited a 6.5 ± 1 µM, >15 µM, which was comparable to or slightly lower in potency than its parent compound, 11*α*,13‐tetrahydrohelenalin acetate (3 ± 0.31 µM). A greater reduction in cytotoxicity was observed for 13‐*S*‐Glutathionyldihydrochamissonolide, which showed >15 µM compared with 2.3 ± 0.15 µM for chamissonolide [59]. Collectively, these findings demonstrate that thiol conjugation can attenuate SL cytotoxicity by competing with protein alkylation; however, the extent of protection depends on the electrophilic centre, reaction kinetics, reversibility of the thiol adduct, and the intracellular thiol environment.

Synergistic ROS‐mediated cytotoxicity has also been demonstrated in combination therapies. Saligraviolide A (Sal A) (**18**) and iso‐seco‐tanapartholide (TNP) (**19**), which show limited anticancer activity when used individually, exhibit pronounced cytotoxic effects when combined [64, 97]. In DLD‐1, HCT‐116 human colon cancer cell lines, individual treatment with Sal A (8 µg/mL) and TNP (10 µg/mL) resulted in approximately 20% reduction in cell viability, whereas combined treatment with Sal A (3 µg/mL): TNP (5 µg/mL) reduced cell viability by 80% [64]. This enhanced effect was attributed to excessive ROS generation and disruption of mitochondrial membrane potential, a response not observed with individual compounds [64].

Additionally, 8‐ 8‐*α*‐erucyl‐1*α*‐hydroxyl hirustinolide (**20**) and 1*α*‐hydroxyhirustinolide‐13‐O‐acetate (**21**) isolated from *Cyanthillium cinereum (L.)*, belonging to the hirustinolide class of the sesquiterpenes, exhibited significant cytotoxicity against 786‐O renal carcinoma cells, MCF‐7 leukaemia and MCF‐7 breast cancer cells with IC_50_ values of 786‐O: 12.5 µg/mL, MCF‐7: 12.8 µg/mL [67]. Their anticancer activity was associated with the overproduction of ROS and the inhibition of DNA replication during the S phase of the cell cycle [67].

### 3.3 Induction of Apoptosis via Intrinsic and Extrinsic Pathways

Apoptosis is a regulated form of programmed cell death, and mitochondrial signalling plays a crucial role in the initiation, making apoptotic pathways attractive targets for anticancer therapy [98]. Proapoptotic agents that disrupt mitochondrial membrane integrity increase membrane permeability, leading to the release of cytochrome c into the cytoplasm [99, 100]. Cytoplasm c subsequently associates with apoptotic activating factor‐1 (Apaf‐1) to form the apoptosome, which activates initiator caspase‐9, followed by effector caspase‐3, culminating in apoptotic cell death [101].

The mitochondrial (intrinsic) apoptotic pathway is activated by multiple intracellular stress signals, including oxidative stress, DNA damage, endoplasmic reticulum stress, cytoskeleton disruption, and inhibition of macromolecular synthesis [102]. These stimuli promote mitochondrial outer membrane permeabilisation and cytochrome c release, leading to apoptosome formation and activation of caspase‐9 and caspase‐3 [103, 104, 105, 106]. Caspase activation features of apoptosis, such as DNA fragmentation, chromatin condensation, and loss of plasma membrane symmetry [107, 108, 109]. In contrast, the extrinsic apoptotic pathway is initiated by ligand‐mediated activation of the death receptor, leading to the activation of caspase‐8 [110].

Several sesquiterpenes lactones induce apoptosis through these pathways. Alantolactone (**15)** triggers mitochondrial‐mediated apoptosis in MDA‐MB‐231 cells by increasing the Bax/Bcl‐2 ratio and promoting cytochrome c release from mitochondria into the cytoplasm [92, 110]. Similarly, combined treatment with Sal A and iso‐seco‐TNP activates caspase‐9, thereby engaging the intrinsic apoptotic pathway [64]. Parthenolide, which contains an *α*‐methylene‐*γ*‐lactone moiety that acts as a Michael acceptor, exerts its anticancer activity through covalent interactions with protein thiol groups and intracellular GSH, leading to GSH depletion and increased ROS generation [63]. In SK‐J1 cells, parthenolide induces G2/M cell cycle arrest and, at higher concentrations (5‐10 µM), causes DNA fragmentation at lower concentrations (0‐2.5 µM), apoptosis occurs without detectable DNA fragmentation but is accompanied by a threefold increase in ROS levels and upregulation of GADD153 mRNA, a gene associated with growth arrest and DNA damage‐induced apoptosis [63].

### 3.4 Cell Cycle Arrest Induced by Sesquiterpene Lactones

Sesquiterpene lactones exert anticancer effects by disrupting normal cell cycle progression, resulting in cell cycle arrest and the subsequent induction of apoptosis [111]. SLs commonly induce arrest at G2/M phase through activation of p53 and inhibition of cyclin‐dependent kinases (CDK), particularly Cdk2, or at the G1/S transition via modulation of cyclin and CDKs [76]. Such cell‐cycle perturbation prevents cell proliferation and sensitises cancer cells to apoptotic signalling.

Deoxyelephantoin (**22**) isolated from *Elephantopus scaber*, exhibits potent cytotoxic activity against the TS/A mammary carcinoma cell lines, with IC_50_ values ranging from 2.9 to 5.8 µM [112]. Its mechanism involves a reduction in DNA content during the G0/G1 phase, increased levels of inactive phosphorylated (Tyr15) Cdk21, downregulation of cyclin B1, and upregulation of the CDK inhibitor p21^waf1/cip1^. In addition, deoxyelephantopin suppresses matrix metalloproteinase‐9 activity and inhibits NF‐*κ*B signalling, further contributing to its anticancer effect [112]. Isoalantolactone (**23**) also exhibits significant cytotoxicity against HeLa cervical cancer cells, with an IC_50_ of 8.15 ± 1.16 µM. Induces G2/M cell cycle arrest, accompanied by phosphatidylserine externalisation, activation of the caspases‐3 and ‐8, and depolarisation of the mitochondrial membrane, collectively leading to apoptotic cell death [66].

### 3.5 Mitogen‐activated Protein Kinase (MAPK) Signalling Pathway

Mitogen‐activated protein kinase (MAPK or MEK) signalling cascade plays a crucial role in regulating cell survival, proliferation, differentiation, and apoptosis [113, 114]. The MAPK pathway is initiated by the activation of RAS, which recruits and activates RAF, leading to phosphorylation of MAPK/ extracellular signal‐regulated kinase (ERK), MEK1 and MEK2 [115, 116]. Activated MEK subsequently phosphorylates ERK, a key effector that controls transcription, translation, lipid metabolism, protein acetylation, and cell cycle progression (Figure 6) [116, 117].

**FIGURE 6:**
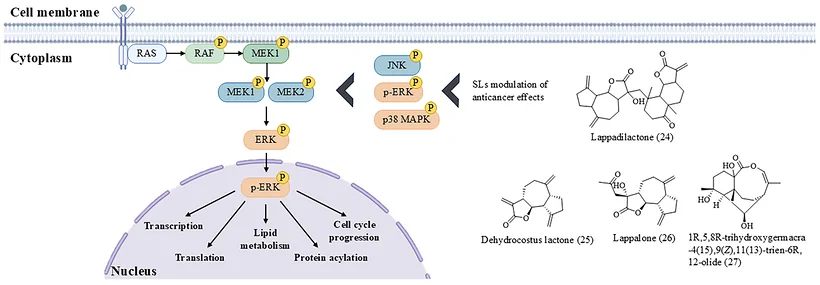
Mitochondria‐mediated apoptosis induced by sesquiterpene lactones: ROS generation and cytochrome‐c release triggered by parthenolide and alantolactone (MEK, mitogen‐activated protein kinase; ERK, extracellular signal‐regulated kinase).

In addition to ERK, other MAPK proteins, including JNK and p38 MAPK, are involved in stress responses and apoptosis. SLs modulate these pathways to exert anticancer effects [64]. For instance, treatment with a combination of Sal A and iso‐seco‐TNP resulted in a significant increase in phosphorylated MAPKs, with p‐JNK, p‐ERK, and p‐p38 levels elevated by 2.5‐, 2‐, and 2‐fold, respectively, after 8h of treatment. ERK and p38 activation required the combined treatment, whereas p‐JNK activation was primarily dependent on iso‐seco‐TNP [64].

Several sesquiterpenes lactones isolated from *Saussaurea lappa*, including lappadilactone (**24**), dehydrocostus lactone (**25**), costunolide (**6**), and lappalone (**26**), showed cytotoxic activity against HeLa, HepG2, and OVCAR‐3 cell lines, with CD_50_ values ranging from 1.6 to 3.5 µg/mL. Among these, lappalone exhibited lower activity, with CD_50_ values exceeding >100 µg/mL across the three tested cell lines [48].

Germacane‐type sesquiterpene, 1R,5,8R‐trihydroxygermacra 4(15),9(*Z*),11(13)‐trien‐6R,12‐olide (**27**) isolated from *Gonospermum gomarae*, exhibited significant anticancer activity against HL‐60, U‐937, SK‐MEL‐1, and A549 cell lines [69]. Similarly, dentatin A (**28**) isolated from *Gonospermum fruticosum*, exhibited cytotoxic effects against HL‐60, U‐937 leukaemia cells, SK‐MEL‐1 melanoma cells, and A549 lung cancer cells [118].

### 3.6 Modulation of Pro‐Inflammatory Signalling in Cancer by Sesquiterpene Lactones

Chronic inflammation plays a critical role in tumour initiation, progression, and metastasis by sustaining the activation of inflammatory mediators and signalling pathways [119, 120, 121]. Pro‐inflammatory cytokines, including interleukin‐6 (IL‐6), interleukin‐1*β* (IL‐1*β*), and TNF‐*α*, along with transcription factors such as NF‐*κ*B, are key contributors to cancer‐associated inflammation [119, 120, 121].

Helenalin is a well‐characterised anti‐inflammatory sesquiterpene lactone that exerts its activity through the covalent alkylation of the sulfhydryl group in the p65 subunit of NF‐*κ*B, thereby inhibiting NF‐*κ*B‐dependent transcription [49]. Its derivatives, helenalin isobutyrate **(40)**, exhibit comparable anti‐inflammatory activity [49]. Similarly, mexicanin I (**2**), which contains a trans‐configured lactone ring, has been shown to inhibit NF‐*κ*B signalling through a related mechanism [49]. Budlein A (**41**) exhibits potent anti‐inflammatory activity by suppressing carrageenan‐induced mechanical hypernociception and reducing the release of TNF‐*α*, keratinocyte‐derived chemokine (KC), and IL‐1*β* [122].

Neurolenin B (**42**), lobatin A (**43**), and lobatin B (**44**), isolated from *Neurolaena lobata*, exhibited strong anti‐inflammatory activity at 10 µM by significantly decreasing IL‐8 secretion in lipopolysaccharides (LPS)‐stimulated endothelial cells and downregulating IL‐8 protein expression in TNF‐*α*‐induced endothelial cells [123]. In addition, SLs isolated from *Siegesbeckia glarescens* exhibit dose‐dependent anti‐inflammatory activity by inhibiting LPS‐induced nitric oxide (NO) and prostaglandin (PGE_2_) production, which is associated with the downregulation of inducible nitric oxide synthase (iNOS) and cyclooxygenase (COX‐2) expression in RAW 264.7 murine macrophage [124]. Furthermore, eudesmane and guaiolide‐type SLs, including Verlotolide C, D and 3‐chloro‐4‐hydroxyguaia‐10 (14),11(13)‐dien‐6,12‐olide, exhibit significant anti‐inflammatory activity by suppressing NO production in the RAW 264.7 macrophage cell line [125]. These findings have a strong potential for SLs as modulators of inflammatory signalling pathways relevant to cancer and inflammatory disorders (Table 3).

**TABLE 3:** Anti‐inflammatory activities of sesquiterpene lactones: mechanistic insights and cellular targets.

Sr. no.	Compound	Class	Source	Cell line	Concentration	Pathway	Mode of inhibition	Ref.
**1**.	Helenalin (1)	Pseudoguaianolide	*A. Chamissonis ssp. folisa*	Jurkat T cells	1–10 µM	NF‐*κ*B	Direct covalent binding with the cysteine residue in the p65 subunits of NF‐*κ*B. Inhibited NF‐*κ*B‐dependent gene expression.	[49]
**2**.	Neurolenin B (42)	Germacranolide	*Neurolaena lobata (L.)*	HUVECtert	1–10 µM	NF‐*κ*B	Decreased LPS‐induced secretion of IL‐8. Decreased secretion of IL‐8 in TNF‐*α*‐stimulated endothelial cells	[123]
**3**.	Lobatin A (43)	Germacranolide	*Neurolaena lobata (L.)*	HUVECtert	1–10 µM	NF‐*κ*B	Decreased LPS‐induced secretion of IL‐8. Decreased secretion of IL‐8 in TNF‐*α*‐stimulated endothelial cells	[123]
**4**.	Lobatin B (44)	Germacranolide	*Neurolaena lobata (L.)*	HUVECtert	1–10 µM	NF‐*κ*B	Decreased LPS‐induced secretion of IL‐8. Decreased secretion of IL‐8 in TNF‐*α*‐stimulated endothelial cells	[123]
**5**.	8*β*‐ isovaleryloxy‐9‐acetoxy‐calculatolide	Germacranolide	*Neurolaena lobata (L.)*	HUVECtert	1–10 µM	NF‐*κ*B	Decreased LPS‐induced secretion of IL‐8 Decreased secretion of IL‐8 in TNF‐*α*‐stimulated endothelial cells	[123]
**6**.	Verlotolide C, D	Eudesmane	*A. verlotorum*	RAW 264.7 macrophage	10 µM	—	Inhibitory activity against LPS‐induced NO production	[125]
**7**.	3‐ chloro‐4‐hydroxyguaia‐10(14),11(13)‐dien‐6,12‐olide	Guainolides	*A. verlotorum*	RAW 264.7 macrophage	10 µM	—	Inhibitory activity against LPS‐induced NO production	[125]

### 3.7 Clinical Advances of Plant‐Derived Sesquiterpene Lactones

Several sesquiterpene lactones are currently being investigated in clinical trials for their potent anticancer activity. Thapsigargin (**29**), isolated from *Thapsia garganica*, exhibits cytotoxicity against multiple cancer cell types, including melanoma, insulinoma, neurons, and breast cancer cells [126, 127, 128]. Thapsigargin causes apoptosis by inhibiting the Sarco‐endoplasmic reticulum CA^2+^ ATPase (SERCA) [129]. Mipsagargin (G‐202), a PSMA‐activated derivative, has been evaluated in phase II trials for hepatocellular carcinoma and developed from thapsigargin by the peptide sequencing (Asp‐Glu‐Glu‐Glu‐Glu) [130, 131]. Parthenolide has also been reported for its potent cytotoxic effect in several preclinical trials [132].

A phase I dose‐escalation study of *Artemisia annua* in patients with advanced ovarian cancer has been completed, with phase II underway to establish optimal dosing (NCT00764036) [133]. The micheliolide derivative ACT001 has entered clinical trials for brain cancers, exhibiting high tolerability and potential clinical benefits. Dimethylamino‐micheliolide was well tolerated with no dose‐limiting toxicities in a dose‐escalation study for glioblastoma (ACTRN12616000228482) [134]. Ongoing studies are assessing its efficacy alone or in combination with pembrolizumab, radiotherapy, or temozolomide (NCT05053880, ChiCTR2000037739).

Arglabin, a farnesyltransferase inhibitor, has been tested as monotherapy or in combination with chemotherapy for various cancers (Targeting RAS oncogene farnesylation is a promising approach for anticancer therapy). In a clinical trial, Arglabin, combined with doxorubicin and cyclophosphamide (CN‐02296454), reduced H‐RAS levels, while Arglabin monotherapy was more effective (CN‐01430638) [135, 136].

## 4 Chemical Diversification of Sesquiterpene Lactones Toward Improved Cytotoxic and Antitumor Activity

Sesquiterpene lactones are widely isolated from plants and have attracted the attention of many scientists due to their anticancer activity, which is primarily attributed to the presence of the *α*‐methylene‐*γ*‐lactone moiety as a pharmacophore [137]. Despite their promising bioactivity, limitations such as moderate potency, selectivity, and bioavailability have encouraged the development of semisynthetic derivatives to enhance their anticancer potential [137]. Structural modification of SLs has emerged as an effective strategy to improve cytotoxicity, lipophilicity, and target specificity while retaining the key electrophilic moiety responsible for biological activity [137].

### 4.1 Acylated and Silylated Derivatives of Hydroxylated Sesquiterpene Lactones

The hydroxy group present in the SL framework provides accessible sites for chemical derivatisation, leading to the formation of acylated and silylated derivatives [138]. For instance, cumanin, isolated from *Ambrosia tenuifolia*, which possesses hydroxy groups at C‐7 and C‐8 positions, was subjected to diacylation and silylation to generate a series of derivatives with altered physicochemical properties [138]. Similarly, helenalin and hymenin were isolated from *Gaillardia megapotamica var*. and *Gaillardia megapotamica Spr*. They were modified using various acylating and silylating agents, yielding derivatives that exhibited a modified biological profile compared to the parent [138]. These modifications primarily aim to enhance lipophilicity and cellular uptake while preserving anticancer activity (Figure 7).

**FIGURE 7:**
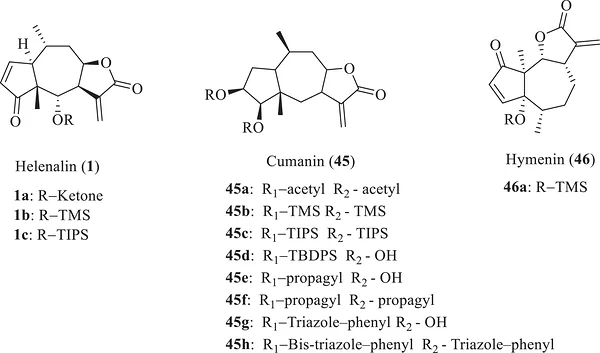
Generalized cumanin, helenalin, and hymenin sesquiterpene lactone scaffolds showing R^1^/R^2^ substitution in cumanin and R‐only modification in helenalin and hymenin.

Oxygenated derivatives of helenanin **(1b, 1c),** cumanin **(45c‐e)**, and hymenin **(46a)** were more active across A549, HBL100, HeLa, SW1573, T47‐D, and WiDr cell lines, with a GI_50_ value of 0.15‐6.8 µM. Silylated derivatives of helenalin compound 1b, 1c were most potent, exhibiting a GI_50_ value of 0.15–0.59 µM, indicating a positive correlation between increased lipophilicity and in vitro cytotoxicity [138]. Bis‐triazole derivative of cumanin **(45h)**, which showed an approximately tenfold increase in potency as compared to cumanin. Hymenin derivative **(46a)** exhibits enhanced antiproliferative activity relative to hymenin [138]. In contrast, incorporation of triazole **(45g, 45h)** into cumanin produced the most pronounced improvement in selectivity and therapeutic window by increasing structural complexity. Among these derivatives, acylated, propylated, and partially functionalized cumin derivatives **(45a–f)** have lower to moderate cytotoxicity compared to the highly active silylated helenalin derivatives **(1b and 1c)** and bis‐triazole derivative of cumanin **(45h)**.

The authors proposed that increased lipophilicity may contribute to this enhanced in vitro cytotoxicity, potentially by facilitating membrane partitioning and cellular uptake. However, this interpretation should be regarded as an SAR hypothesis because the study did not directly establish that the silylated derivatives exhibited superior aqueous solubility, metabolic stability, pharmacokinetic behaviour, or oral bioavailability. Thus, the SAR indicates that simply acylating the hydroxyl group does not enhance cytotoxicity potency, whereas appropriately hydrophobic or heteroaromatic substituents can alter potency and selectivity.

### 4.2 Novel Spiro‐Isoxazoline, Spiroxazolidine, and Spiroaziridine Derivatives of Parthenin

Parthenin possesses both exocyclic and endocyclic double bonds that are susceptible to nucleophilic attack and 1,3‐dipolar cycloaddition. However, due to steric hindrance around the cyclopentenone ring, cycloaddition occurs selectively at the exocyclic olefinic bond [45]. Alkynyl derivatives of parthenin have moderate cytotoxicity compared to the Spiro derivatives. Novel spiro‐isoxazoline, spiroxazoline, and spiroazirine derivatives were prepared through dipolar cycloaddition reaction involving nitrile oxides, nitrones, and azides, respectively [45]. Triazole moiety does not occur in nature, but in pharmaceutical leads, it is a core moiety, and several molecules containing heterocyclic nucleus such as grindelic acid derivatives are reported for the anti‐HIV, anti‐fungal, etc. [139]. The resulting spiro‐isoxazoline derivatives exhibited notable cytotoxic activity, particularly when substituted with electron‐donating or halogenated aromatic moieties, while retaining the essential *α*‐methylene‐*γ*‐lactone pharmacophore [45]. The spiro‐heterocyclic modification is a valuable medicinal chemistry approach for enhancing the anticancer efficacy of sesquiterpene lactones without disrupting their key bioactive structural features.

Parthenin derivatives were evaluated for their anticancer activity against the SW‐620, DU‐145, and PC‐3 cell lines. The spiro derivatives exhibited enhanced cytotoxic activity compared with the parthenin (IC_50_: 38.7–40.3 µM). In the spiroisoxazoline series, compounds **3a, 3b, 3d,** and **3e** showed the highest IC_50_ values of 2.5–6.3 µM, indicating significantly improved potency across all cell lines, whereas compound 3x was weakly active. The spiroisoxazolidine series is the most potent class, with most compounds showing IC_50_ values ranging from 2 to 10 µM, as observed for compounds **3m, 3q, 3r, 3s, 3u,** and **3v**, particularly against SW‐620 and PC‐3 cells; others are weakly cytotoxic (Figure 8). These finding shows that conversion of the exocyclic double olefin into spiro‐fused heterocyclic system can improve the cytotoxic potency of parthenin, although it is mainly depended on the nature and position of the substituents. Simple alkyne‐functionalized derivatives produced only moderate cytotoxicity, whereas conversion of the exocyclic double bond into spiro‐fused heterocycles, particularly spiroisoxazolidine and substituted spiroisoxazoline, resulted in a pronounced increase in potency, indicating that incorporation of appropriately substituted heterocyclic rings is more favourable than simple alkyne modification for enhancing the cytotoxic activity of parthenin. Their effects on the metabolic stability and bioavailability were not established in the study. Therefore, the observed increase in cytotoxicity should be attributed primarily to the structural and electronic consequences of spiro‐heterocycle formation and substituent variation, rather than to presumed improvements in metabolic stability or systemic exposure. Further pharmacokinetic and metabolic studies are required to determine whether these modifications translate into improved metabolic stability, bioavailability, and in vivo anticancer efficacy.

**FIGURE 8:**
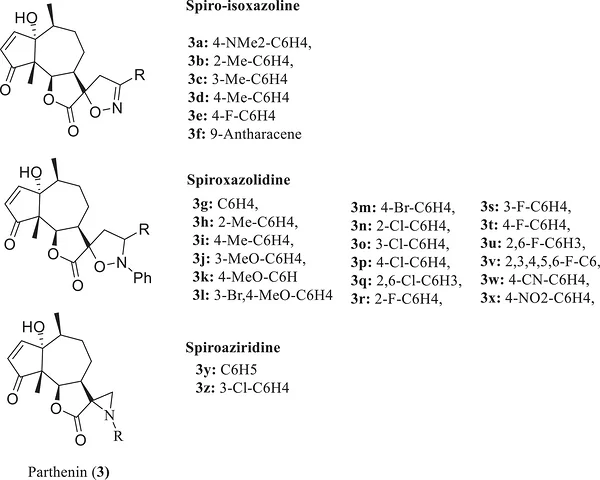
Semisynthetic spiroisoxazoline, spiroisoxazolidine, and spiroaziridine derivatives of parthenin prepared via cycloaddition and ring‐closure strategies.

### 4.3 Two Alkylating Centres, an Ester Derivative of Helenalins and Mexicanin I

The presence of two electrophilic alkylating centres within a single SL molecule has been shown to significantly enhance cytotoxic activity [46]. Derivatives of helenalins and mexicanin I, which contain one alkylating centre at the C‐11 position; the introduction of an additional alkylating centre at the C‐6 position increases reactivity toward biological nucleophiles **(1a‐2c)**, thereby enhancing anticancer potency [46]. Esterification at the C‐6 position of 11*α*,13‐dihydrohelinalin (isovalerate) derivatives further increases lipophilicity, thereby improving cellular permeability [46]. However, the size of the ester group plays a critical role: bulky ester substituents, such as tiglate and isovalerate, reduce cytotoxic activity due to steric hindrance that limits access to the exocyclic methylene group [46]. In contrast, the parent alcohol at the C‐6 position exhibits lower cytotoxicity, underscoring the importance of both steric and electronic factors in optimising biological activity [46]. increases the compound's lipophilicity (Figure 9).

**FIGURE 9:**
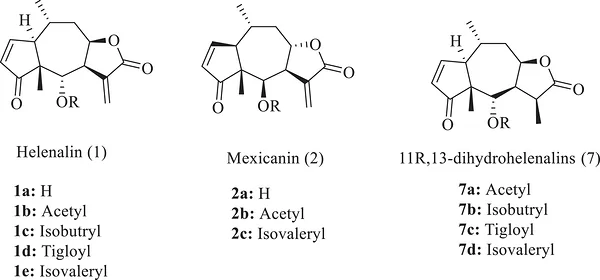
Helenalin, mexicanin I, and 11β,13‐dihydrohelenalin and their corresponding O‐acyl semisynthetic derivatives.

### 4.4 Thiol Derivatives of Alantolactones

Thiol derivatives of alantolactone were synthesised by using the Michael ion reaction targeting the *α*‐methylene‐*γ*‐lactone moiety [140]. In this approach, alantolactone was reacted with various thiols in methanol in the presence of triethylamine. Thiol‐substituted derivatives were evaluated for biological activity in the RAW 246.7 macrophage cell line [140]. These modifications enhance the biological activity of SLs, particularly in pathways associated with inflammation and cancer.

### 4.5 Amine, Diepoxy, Phosphorous‐Containing Derivatives of Arglabin

Arglabin contains an exomethylene group conjugated to a *γ*‐lactone carbonyl [141]. Amination of arglabin in an alcohol medium, proceeding chemoselectively through an activated double bond to synthesise a series of amino derivatives [142]. Additionally, pyrazole derivatives were synthesised via a reaction involving ethylazidoacetate and diazomethane in the presence of CuI. Phosphorylation of arglabin was achieved using dimethyl, diethyl, and dipropyl phosphites, yielding the corresponding phosphonates [142]. Furthermore, chlorhydrin derivatives are prepared by modifying the C‐3/C‐4 epoxy group, yielding a diepoxy‐containing analogue [142]. These derivatives demonstrate the growth‐inhibitory activity against transplantable tumours, including guerin carcinoma, P‐388 leukaemia, and sarcoma‐45 [142]. Arglabin is utilised as a versatile scaffold for the development of anticancer drugs.

## 5 Translational and Pharmacokinetic Challenges

A pharmacokinetic (PK) study is very important for the mechanism of pharmaceutical action. Three SLs, 1‐acetoxy‐6*α*‐hydroxyeriolanolide, *β*‐hydroxyalantolactone, and ivangustin, were administered orally and demonstrated rapid absorption, with first‐peak concentrations occurring within 35 min [143]. 1‐acetoxy‐6*α*‐hydroxyeriolanolide reaches to its highest concentration 609.03 ± 165.43 ng/mL in 0.53 ± 0.24h, while both isomers 1*β*‐hydroxyalantolactone and ivangustin achieved their highest concentration 236.74 ± 43.02 and 278.88 ± 28.49 ng/mL, respectively, at 0.19 ± 0.07 h [143]). This indicates the isomers have higher lipophilicity, allowing more rapid membrane penetration. The double‐peak phenomenon in these compounds results from enterohepatic circulation and reabsorption. 1‐acetoxy‐6*α*‐hydroxyeriolanolide showed a significantly shorter elimination half‐life (9.65 ± 1.43 hr) than both isomers (14.88 ± 0.82 hr and 13.93 ± 2.74 h) [143]). This shorter half‐life indicates 1‐acetoxy‐6*α*‐hydroxyeriolanolide is more readily metabolised and eliminated from the system. absorption efficiency for the isomers (134.82 and 130.31 ng h/mL) versus 1‐acetoxy‐6*α*‐hydroxyeriolanolide (45.94 ng h/mL) [143]). This suggests better bioavailability despite lower absolute concentrations, indicating that the isomers are absorbed more effectively relative to their administered doses.

The compound with a 4‐hydroxy‐2‐methylenebutanoyloxy side chain shows better Caco‐2 permeability than the other structures [144]. Larger, more complex molecules show poor permeability [144]. An additional hydroxyl group, such as 8*α*‐hydroxy‐11*β*,13‐dihydro‐4‐epi‐sonchucarpolide, increases aqueous solubility but decreases permeability due to increased polarity [144]. Modified side with a second alkylating site could optimise pharmacokinetic profiles while maintaining antimicrobial activity [144].

Several studies conclude that costunolide and dehydrocostus lactone exhibit similar actions and PK parameters [145]. Costunolide T_max_ is increased from 9h to 12h, and dehydrocostus lactone from 6h to 12h in vivo due to the other terpenes present in the *Aucklandia lappa* extract and Weichang'an Pills. Apart from this, it could also bind to the human serum albumin through the Van der Waals force and the hydrogen bond [146]. This delayed absorption pattern was observed when comparing the pure compound with the traditional Chinese medicine [145]. Clinical translation of this class of compounds is difficult due to the poor aqueous solubility, non‐selective binding to the undesired target as a Michael acceptor [147]. The prodrug approach increases the aqueous solubility of SLs by the addition of the amine into the *α*‐methylene‐*γ*‐butyrolactone [148]. Nanoparticle delivery improves the delivery of low‐aqueous‐soluble, poorly stable, and orally bioavailable phytochemicals [149]. Nanocarrier (polymeric or lipid nanoparticles) and mucoadhesive depot systems for a low aqueous solubility compound like artemisinin [150].

## 6 Conclusion

Sesquiterpene lactones constitute a chemically diverse class of plant‐derived natural products with substantial relevance in anticancer research. Their biological activity is largely governed by key electrophilic functionalities, particularly the *α*‐methylene‐*γ*‐lactone moiety, which enables covalent interaction with critical cellular targets. However, this electrophilic reactivity is a double‐edged pharmacophore, as the same properties that confer potent biological activity may also promote nonspecific protein alkylation, off‐target effects, glutathione depletion, and toxicity. This review highlights the influence of structural features, biosynthetic origins, and structural‐activity relationships on the pharmacological behaviour of sesquiterpene lactones. Mechanistic studies demonstrate that sesquiterpene lactones exert anticancer effects by modulating multiple signalling pathways, including inhibition of NF‐*κ*B, induction of oxidative stress, apoptosis, cell‐cycle arrest, and MAPK regulation.

Advances in biosynthetic insights and semisynthetic derivatisation have expanded opportunities to optimise efficacy and selectivity. Nevertheless, future development should move beyond the simple identification of highly cytotoxic compounds to target‐selective interactions that engage cancer‐relevant targets while minimising nonspecific thiol reactivity and toxicity in normal cells. In addition, semisynthetic modification strategies should be evaluated not only for improvements in in vivo cytotoxicity but also for metabolic stability, bioavailability, the pharmacokinetics‐pharmacodynamics relationship, and systemic exposure.

However, limitations such as poor bioavailability and insufficient clinical validation persist. Future efforts should prioritise rational structural modification, improved delivery strategies, and comprehensive in vivo and clinical studies to facilitate the translation of sesquiterpene lactones into anticancer therapeutics.

## Author Contribution

**Kedar Nimba Chaudhari**: conceptualisation, methodology, data curation, writing – review and editing. **Ragini Yadav**: data curation, writing – review and editing. **Mayank Kashyap**: data curation, writing – review and editing. **Shreyans K. Jain**: conceptualisation, methodology, data curation, validation, supervision, writing – original draft, writing – review and editing.

## Conflicts of Interest

The authors declare no conflicts of interest.

## Data Availability

Data sharing is not applicable to this article as no datasets were generated or analysed during the current study.
